# Early detection of colorectal adenocarcinoma: a clinical decision support tool based on plasma porphyrin accumulation and risk factors

**DOI:** 10.1186/s12885-018-4754-2

**Published:** 2018-08-22

**Authors:** Manuela Lualdi, Adalberto Cavalleri, Luigi Battaglia, Ambrogio Colombo, Giulia Garrone, Daniele Morelli, Emanuele Pignoli, Elisa Sottotetti, Ermanno Leo

**Affiliations:** 1 Medical Physics Unit, Fondazione IRCCS Istituto Nazionale dei Tumori, Via Venezian 1, 20133 Milan, Italy; 2 Epidemiology and Prevention Unit, Fondazione IRCCS Istituto Nazionale dei Tumori, Milan, Italy; 3 Colorectal Cancer Unit, Fondazione IRCCS Istituto Nazionale dei Tumori, Milan, Italy; 4 Health Administration, Fondazione IRCCS Istituto Nazionale dei Tumori, Milan, Italy; 5 Department of Pathology and Laboratory Medicine, Fondazione IRCCS Istituto Nazionale dei Tumori, Milan, Italy; 6 Department of Medical Oncology, Fondazione IRCCS Istituto Nazionale dei Tumori, Milan, Italy

**Keywords:** Colorectal cancer, adenocarcinoma, tumor marker, native fluorescence, Protoporphyrin IX, Coproporphyrin I

## Abstract

**Background:**

An increase in naturally-occurring porphyrins has been described in the blood of subjects bearing different kinds of tumors, including colorectal, and this is probably related to a systemic alteration of heme metabolism induced by tumor cells. The aim of our study was to develop an artificial neural network (ANN) classifier for early detection of colorectal adenocarcinoma based on plasma porphyrin accumulation and risk factors.

**Methods:**

We measured the endogenous fluorescence of blood plasma in 100 colorectal adenocarcinoma patients and 112 controls using a conventional spectrofluorometer. Height, weight, personal and family medical history, use of alcohol, red meat, vegetables and tobacco were all recorded. An ANN model was built up from demographic data and from the integral of the fluorescence emission peak in the range 610–650 nm. We used the Receiver Operating Characteristic **(**ROC**)** curve to assess performance in distinguishing colorectal adenocarcinoma patients and controls. A liquid chromatography-high resolution mass spectrometry (LC-HRMS) analytical method was employed to identify the agents responsible for native fluorescence.

**Results:**

The fluorescence analysis indicated that the integral of the fluorescence emission peak in the range 610–650 nm was significantly higher in colorectal adenocarcinoma patients than controls (*p* < 0.0001) and was weakly correlated with the TNM staging (Spearman’s rho = 0.224, *p* = 0.011). LC-HRMS measurements showed that the agents responsible for the fluorescence emission were mainly protoporphyrin-IX (PpIX) and coproporphyrin-I (CpI). The overall accuracy of our ANN model was 88% (87% sensitivity and 90% specificity) with an area under the ROC curve of 0.83.

**Conclusions:**

These results confirm that tumor cells accumulate a diagnostic level of endogenous porphyrin compounds and suggest that plasma porphyrin concentrations, indirectly measured through fluorescence analysis, may be useful, together with risk factors, as a clinical decision support tool for the early detection of colorectal adenocarcinoma. Our future efforts will be aimed at examining how plasma porphyrin accumulation correlates with survival and response to therapy.

## Background

About 90% of people whose colorectal cancer (CRC) is diagnosed before it has spread to nearby lymph nodes or organs (localized stage) survive more than five years after diagnosis. However, only 14% of those whose cancer has spread to distant parts of the body (distant stage) survive five years [1]. CRC progresses slowly from detectable and curable precancerous lesions, so diagnosis at an early stage and removal of clinically significant adenomas aims to reduce the incidence of advanced tumors and hence mortality. Although there is proven evidence that screening reduces the incidence of CRC [2, 3], the widespread diffusion of the most effective examination techniques such as colonoscopy and sigmoidoscopy is limited by the availability of resources and by low compliance. Acceptable and inexpensive filter tests should boost the numbers of people who undergo regular screening and should select for colonoscopy or sigmoidoscopy those who are most likely to benefit.

Currently the fecal immunochemical test (FIT) is considered the screening test of choice for CRC: it shows greater sensitivity than the guaiac-based fecal occult blood test (gFOBT) [4, 5], is more specific and less expensive than the FIT-DNA test [4, 6] and has higher specificity and a better positive likelihood ratio than fecal M2-type pyruvate kinase [7]. However, blood tests are likely to be more acceptable than stool tests in population-based screening [8]. Recently several approaches based on antibody signature have been developed for early detection of CRC, but further validation studies are required before they can be proposed in clinical practice [9].

There are several biomolecules which, when excited at suitable wavelengths, give fluorescence emission over a wide spectral range. As a pathological condition develops in tissue, changes occur in biochemical, physicochemical and histological properties at the cellular and tissue levels and the fluorescence emission spectrum may show changes, facilitating the distinction between normal and malignant tissue. This finding has been already applied in the early detection of breast, cervix, colorectal and oral cancer [10–13].

In previous works [14, 15], we investigated the possible use of the endogenous fluorescence of blood plasma for the early detection of colorectal adenocarcinoma, which accounts for 96% of all CRC cases [4]. In these studies, the only parameter used to discriminate between colorectal adenocarcinoma patients and their control counterparts was the intensity of fluorescence at 623 nm, whose overall accuracy in distinguishing the two populations was 73% (80% sensitivity and 50% specificity). Subsequent investigations have suggested extending the fluorescence signal analysis over a wider spectral range and using the risk factors for this pathology together with the results of fluorescence analysis to boost the diagnostic power of the developing marker.

In this pilot study we used the integral of the fluorescence emission peak in the range 610–650 nm, hereafter referred to as IF-INT, and the demographic data of the subjects enrolled to train an artificial neural network (ANN) multiparametric test for colorectal adenocarcinoma. The primary objective was to verify the performance of the classifier for distinguishing patients with colorectal adenocarcinoma from control subjects. The second endpoint was to identify the agents responsible for the fluorescence signal by liquid chromatography-high resolution mass spectrometry (LC-HRMS).

## Methods

### Participants

Between January 2013 and December 2014, we consecutively recruited all colorectal adenocarcinoma patients who accessed the Colon-Rectal Surgery Unit of the Fondazione IRCCS Istituto Nazionale Tumori in Milan, Italy, and who agreed to participate in the study. Patients were eligible if they were more than 18 years old, had never had other tumors, had no concomitant non-tumor gastrointestinal diseases such as Crohn’s disease and diverticulitis, had not undergone chemotherapy in the six months prior to admission and had a histopathologic diagnosis of colorectal adenocarcinoma. A blood sample was taken before surgery from all patients meeting the selection criteria. In the same period, we recruited controls among the patients who entered the Endoscopy Unit of our Institute to undergo colonoscopy for family history, following a positive outcome of FIT or gFoBT or for a bleeding episode that required immediate diagnostic assessment. Subjects were eligible if they were more than 18 years old, had never had cancer, did not have concomitant gastrointestinal diseases such as Crohn’s disease and diverticulitis, and colonoscopy confirmed the absence of colorectal carcinoma, adenomas or inherited syndromes. A blood sample was taken before colonoscopy from all the patients meeting the selection criteria.

All the subjects enrolled gave written informed consent to participate and completed a questionnaire to record information on height and weight, alcohol intake (none, < 1 and ≥ 1 drink/day), red meat (none, 1–2, 3–4 and > 4 portions/week) and vegetables (none, 1 and > 1 portion/day), smoking status (never, former or current smoker) and family history of CRC (yes or no, up to second-degree relatives).

The Ethics Committee of our Institute approved this study protocol before subjects were enrolled.

### Fluorescence measurements

Blood samples were collected in lithium-heparin tubes, centrifuged and the supernatant was collected. All supernatants were stored at − 20 °C until analysis. The fluorescence analysis method has been described in detail previously [14]. Briefly, fluorescence was measured in plasma samples with a conventional spectrofluorimeter (Model F-3000, Hitachi Ltd. Tokyo, Japan), selecting an excitation wavelength of 405 nm and recording the fluorescence emission spectra in the range 430–700 nm. In view of the broad variability in the fluorescence intensity from sample to sample, each fluorescence spectrum was normalized by dividing the fluorescence intensity at each wavelength by the maximum intensity of the spectrum and the IF-INT was finally calculated. The non-parametric Mann-Whitney test for unpaired data was employed to assess differences in the mean IF-INT between colorectal adenocarcinoma patients and control subjects. The Spearman correlation analysis was performed to evaluate the correlation between IF-INT and the TNM-UICC classification of the disease [16]. A *p* value < 0.05 was taken to indicate statistical significance.

The predictive performance of IF-INT was assessed from the area under the receiver operating characteristic curve (AUROC). Sensitivity, specificity, negative predictive value (NPV) and positive predictive value (PPV) were also calculated.

### Artificial neural network

Artificial neural networks are ideal for modeling non-linear relationships between a set of predictors or input variables and one or more responses or output variables. The action of an ANN is defined by the neurons of each layer, which are the basic computational units of the network, and by the connections between the layers, with their weights. Among all the possible network architectures, the feed-forward neural network with back-propagation training has been widely adopted for realistic nonlinear multiple regression in different medical fields [17]. In a feed-forward network, each neuron is connected to all the neurons of the previous and subsequent layers, with no connections between neurons on the same layer [18].

In order to develop a classifier to distinguish colorectal adenocarcinoma patients from controls, we built up a feed-forward ANN model using MATLAB software (The Mathworks Inc., Natick, MA). Seven variables were considered as inputs for the network: IF-INT, body mass index (BMI), calculated by dividing the weight in kilograms by the square of the height in meters, alcohol consumption, red meat intake, vegetables intake, smoking status and family history of CRC. A three-layer structure was used with only one hidden layer; during network instruction, the number of hidden neurons was varied between 1 and 6. The output of our model was between 0 and 1.

The first phase of model development is the training procedure on a defined set of input variables with known output data. The overall population was randomly divided into three subsets, training, validation and test sets, and the back-propagation algorithm with the early stopping procedure was applied; the leave-one-out cross-validation tool was adopted in order to avoid overfitting the data [19]. Multiple runs of the ANN model were done, with the number of hidden neurons ranging between 1 and 6, with random choices for the weights and biases at each cycle and changing the relative composition of the training, validation and test sets. The moment of stopping the training procedure and the final topology of the network were both decided by minimizing on the validation set the mean square error (MSE), which is the measure of how the predicted and actual data differ. Finally, we examined a test set of data completely unknown to the network and evaluated the predictive performance of the ANN model through ROC analysis. Sensitivity, specificity, NPV and PPV were calculated by imposing a cut-off of 0.60 on the ANN output.

After optimizing the ANN model, the relative importance (RI%) of the input variables was assessed through the most squares method [20] to evaluate the role of each predictor in the prediction process.

### Qualitative mass-spectrometric analysis

Several studies suggest that tumor cells are able to produce porphyrins naturally or after administering their precursor [13, 21–23] and that porphyrin compounds are responsible for plasma red fluorescence [24, 25]. On this basis, we assumed that the difference between the blood fluorescence spectra of colorectal adenocarcinoma patients and control subjects was due to endogenous porphyrins accumulated in cancer cells as a result of a systemic alteration of heme metabolism, and then pumped out to plasma. To identify the molecules responsible for the fluorescence signal, we developed an LC-HRMS method to determine the mass-to-charge ratio (M/Z) with 1 ppm error for the substances isolated from the plasma samples. This information, together with the retention time (RT), serves to determine the nature of the substances themselves.

In detail, 1000 μL of ethyl acetate:acetic acid (3:1 *v*/v) were added to 400 μL of plasma; after vortexing and centrifugation at 17000 relative centrifugal force (RCF-g) for 20 min at 15 °C, the supernatant was transferred into a polypropylene tube. The organic layer was evaporated in a stream of nitrogen at 40 °C. The dry residue of plasma samples was reconstituted with 100 μL water: aceto- nitrile:acetic acid 2:1:1 v/v/v and 15 μL were injected into the LC-HRMS system.

Liquid chromatography separation was done with a Dionex Ultimate 3000 RSLC (Thermo Fisher Scientific, Waltham, MA) equipped with an XBridge BEH300-C18 3.5 μm, 150 × 2.1 mm column (Waters Corporation, Milford, MA). A binary mobile phase and gradient elution were used at a flow rate of 350 μL/min. Mobile phases were: A, H_2_O with 10% acetic acid and B, acetonitrile with 10% acetic acid. In the first 6 min, phase B was raised from 40 to 98%; for the next 2 min phase B was kept at 98%; finally, reconditioning to the initial phase composition was scheduled in the last 5 min.

An Orbitrap Elite high-resolution mass spectrometer (Thermo Fisher Scientific, Waltham, MA) was used as detector, working in electro-spray ionization mode in positive polarity: spray voltage was 4.5 kV; sheath and auxiliary gas (nitrogen) pressure were 40 and 15 a.u. respectively; the vaporizer temperature was 320 °C; the capillary temperature was 350 °C; s-lens was 69 V. M/Z values were acquired in full scan (FS) mode (mass range 250–1300 Da); resolving power was set to 120.000 full width at half maximum (FWHM) to permit exact mass extraction of molecular ions from the FS spectra; chromatographic RTs were used to ensure reliable compound identification.

Five analytes were selected that are involved in heme biosynthesis and have characteristic fluorescence emission in the range 610–650 nm: protoporphyrin-IX (PpIX), coproporphyrin-I (CpI), iron protoporphyrin IX (FePpIX), biliverdin (Bv) and protoporphyrin IX dimethyl ester (dmPpIX) (Sigma Aldrich, St. Louis, MO). These analytes, each at a concentration of 1 mg/ml, were used to prepare a standard solution. Twenty plasma samples with and 20 without evidence of native fluorescence, and the porphyrin standard solution, were processed with the analytical method.

## Results

### Participants

From January 2013 to December 2014, 135 patients who accessed the colorectal surgery unit of our Institute were consecutively enrolled. Fifteen were excluded for missing information; 12 were non-eligible because they had received chemotherapy in the six months prior to blood sampling, five patients because their blood sample was hemolyzed and therefore not analyzable; three were excluded after surgery as they were found not to have adenocarcinoma. The colorectal adenocarcinoma patients were classified postoperatively according to the TNM-UICC classification based on clinical and pathological findings. Seven patients were stage 0, 31 stage I, 29 stage II, 25 stage III, and 8 stage IV. In the same period, 157 patients who accessed the endoscopy unit of our Institute were recruited. Thirty-one were excluded as the colonoscopy and the histological exam confirmed the presence of an adenoma, familial adenomatous polyposis or Crohn’s disease; 14 patients were excluded for missing information.

The main demographic characteristics of the study population are summarized in Table 1. Patients and controls were divided into four groups according to their BMI: underweight (< 18.5 Kg/m^2^), normal (18.50–24.99), overweight (25.00–29.99) and obese (≥30.00) [26]. Colorectal adenocarcinoma patients were older than controls, with similar mean ages for women and men in both groups*.* Distributions of BMI, smoking, alcohol and meat consumption by sex indicated a healthier lifestyle for women than men. In addition, differences in BMI and alcohol and meat consumption between colorectal adenocarcinoma patients and control groups were more marked for men than women. Among men, colorectal adenocarcinoma patients were less frequently normal weight and more frequently overweight, had higher alcohol and red meat intake. As regards smoking, there seemed to be no marked differences between patients and controls, of either sex. No significant differences were observed in vegetable intake between colorectal adenocarcinoma patients and controls, or between men and women. Finally, a family history of CRC was definitely more frequent among controls than cases. The controls in our study were in fact pre-selected subjects at risk for whom a colonoscopy had been prescribed, while in the patients the disease had actually manifested itself.

**Table 1:** Main demographic data of the enrolled subjects

Demographic data	Patients (100)		Control subjects (112)	
Gender	Female	Male	Female	Male
	42	58	56	56
Age [y]: median (range)	65 (28–88)	65 (46–88)	56 (19–78)	57 (23–82)
BMI [kg/m^2^]				
< 18.50	1 (2.4%)	2 (3.4%)	3 (5.4%)	0 (0%)
18.50–24.99	25 (59.5%)	23 (39.7%)	37 (66.1%)	31 (55.4%)
25.00–29.99	12 (28.6%)	26 (44.8%)	13 (23.2%)	18 (32.1%)
≥ 30.00	4 (9.5%)	7 (12.1%)	3 (5.4%)	7 (12.5%)
Alcohol consumption [drinks/day]				
none	19 (45.2%)	6 (10.3%)	32 (57.1%)	26 (46.4%)
< 1	13 (31.0%)	17 (29.3%)	13 (23.2%)	11 (19.6%)
≥ 1	10 (23.8%)	35 (60.4%)	11 (19.6%)	19 (34.0%)
Red meat intake [portions/week]				
none	1 (2.4%)	0 (0%)	3 (5.4%)	1 (1.8%)
1–2	15 (35.7%)	13 (22.4%)	27 (48.2%)	29 (51.8%)
3–4	19 (45.2%)	35 (60.4%)	23 (41.1%)	19 (33.9%)
> 4	7 (16.7%)	10 (17.2%)	3 (5.4%)	7 (12.5%)
Vegetables [portions/day]				
none	10 (23.8%)	4 (6.9%)	10 (17.9%)	14 (25.0%)
1	28 (66.7%)	51 (87.9%)	33 (58.9%)	30 (53.6%)
> 1	4 (9.5%)	3 (5.2%)	13 (23.2%)	12 (21.4%)
Smoking				
never smoker	28 (66.7%)	30 (51.7%)	38 (67.9%)	30 (53.6%)
former smoker	5 (11.9%)	17 (29.3%)	7 (12.5%)	14 (25.0%)
current smoker	9 (21.4%)	11 (19.0%)	11 (19.6%)	12 (21.4%)
Family history of CRC				
yes	3 (7.1%)	11 (19.0%)	26 (46.4%)	20 (35.7%)
no	39 (92.9%)	47 (81.0%)	30 (53.6%)	36 (64.3%)

*BMI* body mass index

### Fluorescence measurements

As found in our previous studies [14, 15], the plasma fluorescence emission peaks between 610 and 650 nm of colorectal adenocarcinoma patients and controls differed significantly. The average IF-INT for all patients was significantly higher than the average for all controls: 170.36 ± 58.42 a.u. vs 107.85 ± 34.55 a.u. with *P* < 0.0001. The Spearman’s rho correlation coefficient between IF-INT and TNM staging was 0.224 (*P* = 0.011), suggesting that there is a weak positive linear correlation between IF-INT and disease severity. With the arbitrary cut-off of 125.00 a.u., the overall accuracy of IF-INT in correctly distinguishing between colorectal adenocarcinoma patients and controls was 84%, with 85% sensitivity and 79% specificity. The AUROC was 0.786.

### Artificial neural network

**Fig. 1:**
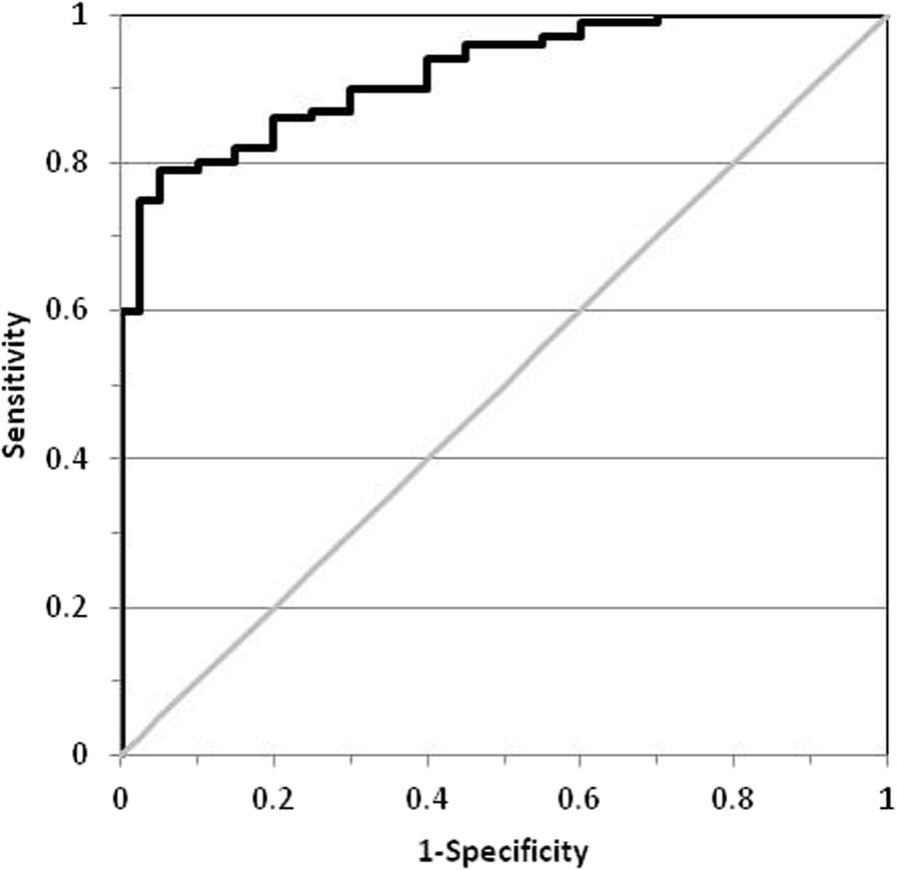
Receiver operating characteristic curve for the optimized artificial neural network classifier based on demographic data and the integral of the fluorescence emission peak in the range 610–650 nm. The area under the ROC curve was 0.828

The optimized ANN architecture was achieved using five hidden neurons; the best validation was reached after 157 iterations, with MSE 0.079 and a coefficient of determination R^2^ of 0.987. Table 2 reports the sensitivity, specificity, NPV and PPV with an arbitrary cut-off of 0.60 on ANN output, for the training, validation and test sets. Figure 1 shows the ROC curve for the test set; the resulting AUROC was 0.828. Table 3 reports the relative importance and rank of the input variables.

**Table 2:** Performance of the artificial neural network classifier in differential diagnosis of colorectal adenocarcinoma in patients and controls

	Training set	Validation set	Test set
Total population	146	33	33
Sensitivity	88.9%	84.6%	86.7%
Specificity	91.9%	85.0%	88.9%
NPV	89.5%	89.5%	88.9%
PPV	91.4%	78.6%	86.7%

*NPV* Negative Predictive Value*, PPV* Positive Predictive Value

**Table 3:** Relative importance and rank of the input variables of the artificial neural network classifier

	Relative importance	Rank
IF-INT	78.73%	1
BMI	10.22%	2
Family history for CRC	5.01%	3
Red meat intake	4.04%	4
Alcohol consumption	1.30%	5
Smoking	0.45%	6
Vegetable intake	0.25%	7

*BMI* body mass index

### Qualitative mass-spectrometric analysis

Table 4 reports the chromatographic and spectrometric properties of the five porphyrin compounds mainly involved in heme biosynthesis, used to verify their presence in the plasma samples submitted to fluorescence analysis. Theoretical M/Z ratios are shown, with the experimental M/Z ratios and the RTs from the analytical method.

**Table 4:** Chromatographic and spectrometric properties of the analytes involved in heme biosynthesis

Porphyrin	Molecular formula	Theoretical M/Z ratio [M + H]^+^	Experimental M/Z ratio [M + H]^+^	RT [min]
Protoporphyrin IX	C_34_H_34_N_4_O_4_	563.2653	563.2651	6.88
Iron protoporphyrin IX	C_34_H_32_FeN_4_O_4_	616.1768	616.1775	4.48
Biliverdin	C_33_H_34_N_4_O_6_	583.2551	583.2565	2.96
Coproporphyrin I	C_36_H_38_N_4_O_8_	655.2762	655.2775	1.85
Protoporphyrin IX dimethylester	C_36_H_38_N_4_O_4_	591.2966	591.2961	8.06

*M/Z* Mass to charge ratio, *RT* Retention time

**Fig. 2:**
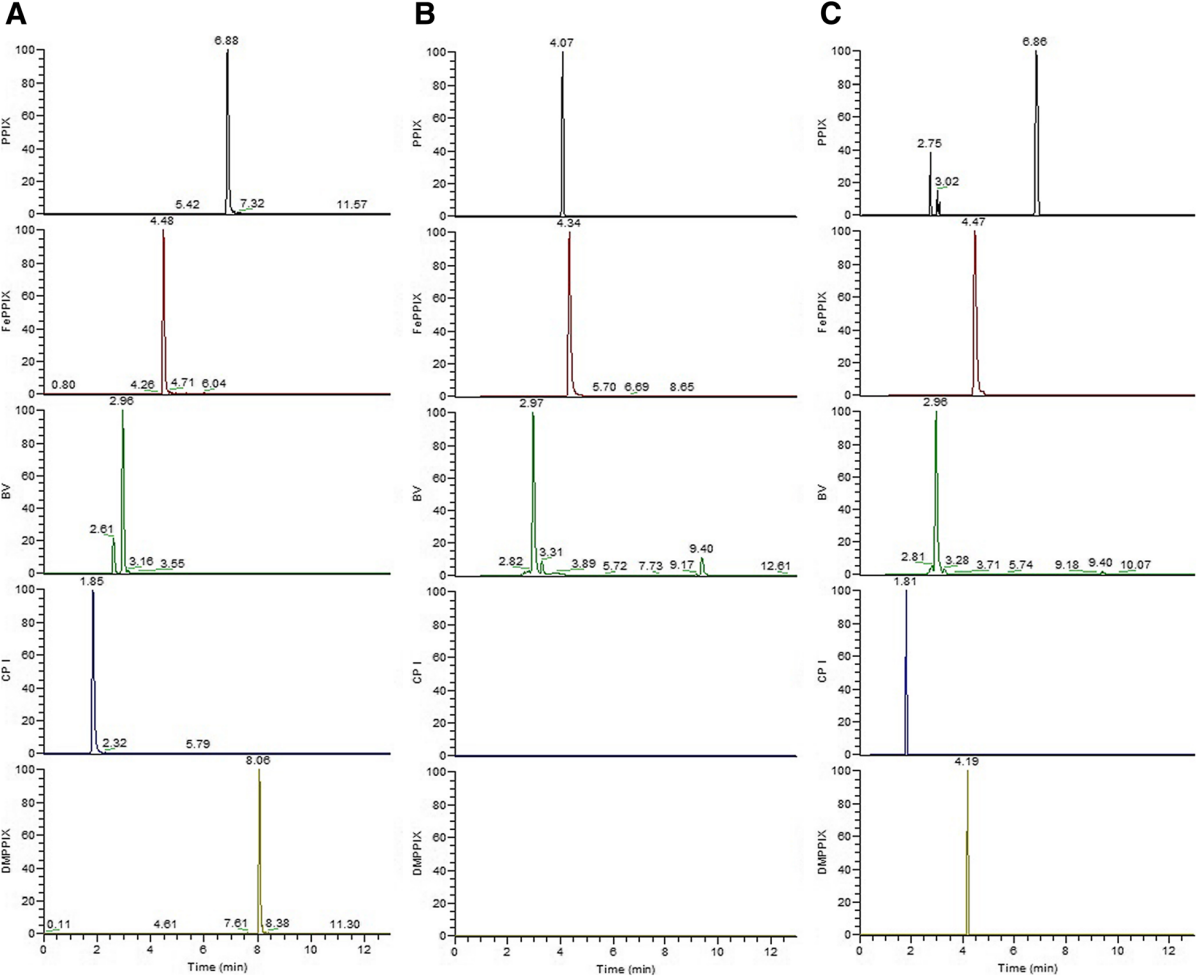
LC-HRMS chromatograms of: (**a**) standard solution of protoporphyrin IX (PpIX), iron protoporphyrin IX (FePpIX), biliverdin (Bv), coproporphyrin I (CpI) and protoporphyrin IX dimethyl ester (dmPpIX); (**b**) plasma sample of a control subject with low fluorescence emission in the range 610–650 nm; (**c**) plasma sample of a colorectal adenocarcinoma patient with high fluorescence emission in the range 610–650 nm

Figure 2 shows typical chromatograms (relative abundance vs retention time) of the extracted exact mass of the analytes: A, porphyrin standard solution (reference chromatogram); B, plasma sample of a control subject with low fluorescence emission in the range 610–650 nm; C, plasma sample of a CRC patient with high fluorescence emission in the same range. The peaks shown in Fig. 2 with a non-tabulated RT correspond to substances not contained in the porphyrin standard solution. Verification of the cross-correspondence of the M/Z values and RTs enabled us to identify the analytes responsible for the native fluorescence of plasma. Protoporphyrin IX and coproporphyrin I were the only analytes present only in the patients’ plasma samples and not in those of the controls. The other analytes were non-specific: biliverdin and iron protoporphyrin IX were present in the plasma samples of both patients and controls, while protoporphyrin IX dimethylester was not found in either case.

## Discussion

In tumor cells the synthesis of PpIX is highly activated and ferrochelatase mRNA expression is down-regulated so PpIX tends to accumulate specifically in tumor tissues [27, 28]. Treatment with 5-aminolevulinic acid (5-ALA) results in progressive accumulation of PpIX in malignant tissue but not in the surrounding tissue, thus offering a means of distinguishing healthy from pathological tissues, exploiting the fluorescence properties of PpIX [29]. Several studies have demonstrated the broad applicability in cancer detection of fluorescence analysis of intrinsic or stimulated PpIX, on samples of ex vivo tissue and biofluids in which the PpIX may pour out of the tumor cells [21–25, 30].

In this study we examined the potential utility of the plasma concentrations of intrinsic porphyrin compounds in the early diagnosis of colorectal adenocarcinoma. We found significantly higher endogenous porphyrin concentrations in the plasma of colorectal adenocarcinoma patients than control subjects; this was indirectly evident from analysis of the native fluorescence spectrum and directly confirmed by LC-HRMS analysis. Fluorescence analysis is a very sensitive technique, easy and quick to implement but it cannot precisely determine the agent responsible for the fluorimetric signal, since different compounds may have the same fluorescence spectrum. LC-HRMS is much more laborious and expensive but does determine the agents responsible for the fluorescence signal by calculating the mass of the substances in the sample.

In the presented method, the analytical descriptor selected to distinguish between colorectal adenocarcinoma patients and control subjects is the integral of the fluorescence emission peak between 610 nm and 650 nm, acquired with a conventional fluorimeter following blue light irradiation. According to our LC-HRMS analysis, the agents responsible for plasma fluorescence emission in the selected wavelength range were mainly protoporphyrin-IX and coproporphyrin-I. Both these compounds have fluorescence emission in the range 610–650 nm when excited at 405 nm, with maximum emission wavelengths at respectively 622 and 632 nm [31]. Investigations currently in progress might clarify whether one or both porphyrins are accumulated by cancer cells more than by healthy ones.

About 75% of CRC patients have sporadic forms of the disease. The remaining 25% have a family history of colorectal cancer, adenomatous polyps or inherited syndromes such as familial adenomatous polyposis (FAP) and Lynch syndrome, suggesting a contribution of inherited genes, shared environmental factors, or some combination of these [1, 4, 32]. Other CRC risk factors include overweight, especially having a larger waistline, type 2 diabetes, physical inactivity, a diet that is high in red and processed meats and poor in vegetables and fruits, smoking, heavy alcohol use, older age, a personal history of adenomatous polyps and inflammatory bowel disease, including ulcerative colitis or Crohn’s disease [4, 32–34]. A mathematical predictive model that integrates risk factors and the result of one or more tumor markers could potentially enhance the diagnostic performance of the markers themselves by simulating the diagnostic process of a physician who simultaneously evaluates the results of laboratory tests and the patient’s personal and family medical history. ANNs offer a relatively new method for predictive modeling in medicine, and are used to map and predict outcomes in complex relationships between given ‘inputs’ (e.g. risk factors, laboratory test results, morphological findings from radiological examinations) and sought-after ‘outputs’ (classification or diagnosis) [17, 35–37]. In contrast with traditional statistical techniques, ANNs are capable of automatically resolving these relationships without the need for a priori assumptions about the nature of the interactions between variables; they employ various statistical, probabilistic and optimization techniques that enable computers to learn from examples and to detect hard-to-discern patterns from large, noisy or complex data sets. The ANN model we describe is a clinical decision support tool based on plasma porphyrins accumulation and risk factors. In this preliminary study, the pre-selection of the enrolled subjects did not permit us to consider some risk factors for this pathology, for example age. Furthermore, the personal history of chronic gastrointestinal diseases was not taken into consideration because we did not know a priori how these pathologies might interact with the plasma native fluorescence spectrum. Our future efforts will focus on extending the study to a larger cohort of subjects in which to consider all the possible risk factors.

We found the overall accuracy of IF-INT for colorectal adenocarcinoma detection was 84%, with 85% sensitivity and 79% specificity, while the overall accuracy of the ANN model (IF-INT plus risk factors) was 88%, with 87% sensitivity and 90% specificity. Although the relative importance of each individual risk factor is significantly lower than that of porphyrin concentration, the contribution of risk factors is not irrelevant in the diagnostic performance of the ANN model and could hopefully be increased considering other risk factors currently unverifiable on the our study population. Accuracy, sensitivity and specificity of our ANN model are comparable to the performances of FITs for colorectal cancer detection, as reported in a meta-analysis performed by Lee et al. [38] that suggests that the pooled sensitivity and specificity of FITs were approximately 79% and 94%, respectively, with an overall accuracy of 95%. FIT sensitivity may decrease with any delay in processing the sample and, like for all stool-based tests, compliance is lower than with blood-based analysis. To appreciate the actual predictive performances of IF-INT and of our ANN model in comparison to FIT, we are planning a comparative study using colonoscopy as the gold standard to assess the two markers on the same population of colorectal adenocarcinoma patients and control subjects.

## Conclusion

The results reported in our paper confirm the presence of diagnostic levels of endogenous porphyrin compounds in the blood plasma of colorectal adenocarcinoma patients and suggest that the measurement of plasma porphyrins concentration may be applied, together with risk factors, as a clinical decision support tool for the early detection of colorectal adenocarcinoma. Further investigations of selected CRC patients are under way to assess IF-INT’s utility as a marker that correlates with survival and response to therapy.

## Abbreviations

ANN: Artificial neural network
AUROC: Area under receiver operating characteristic curve
BMI: Body mass index
Bv: Biliverdin
CpI: Coproporphyrin-I
CRC: Colorectal cancer
dmPpIX: Protoporphyrin IX dimethyl ester
FePpIX: Iron protoporphyrin IX
FIT: Fecal immunochemical test
FS: Full scan
FWHM: Full width at half-maximum
gFOBT: Guaiac-based fecal occult blood test
IF-INT: Integral of the fluorescence emission peak
LC-HRMS: Liquid chromatography-high resolution mass spectrometry
MSE: Mean square error
NPV: Negative predictive value
PpIX: Protoporphyrin-IX
PPV: Positive predictive value
ROC: Receiver operating characteristic curve
RT: Retention time
